# Sphingosine Kinase 1 urothelial expression is increased in patients with neurogenic detrusor overactivity

**DOI:** 10.1590/S1677-5538.IBJU.2014.0676

**Published:** 2015

**Authors:** Quentin Ballouhey, Jalesh N. Panicker, Catherine Mazerolles, Mathieu Roumiguié, Falek Zaidi, Pascal Rischmann, Bernard Malavaud, Xavier Gamé

**Affiliations:** 1 Département d'Urologie, CHU Rangueil, Toulouse, France; 2 Department of Uro-Neurology, UCL Institute of Neurology, The National Hospital for Neurology and Neurosurgery, Queen Square, London, WC1N ^3^BG, United Kingdom; 3 Laboratoire d'Anatomo-pathologie, CHU Rangueil, Toulouse, France; 4 INSERM I2MC UMR1048, CHU Rangueil, Toulouse, France 1 Département d'Urologie, CHU Rangueil, Toulouse, France

**Keywords:** Urinary Bladder, Neurogenic, Urinary Bladder, Overactive, sphingosine kinase [Supplementary Concept], Urothelium, Immunochemistry

## Abstract

**Objectives:**

To evaluate the expression of sphingosine kinase 1 (SPK1) in the bladder wall in patients with neurogenic lower urinary tract dysfunction and its association with clinical, urodynamic and pathological features.

**Materials and Methods:**

The expression of SPK1 was studied in bladder wall specimens obtained from cystectomy using immunohistochemistry in ten patients with spinal cord injury (n=8) or multiple sclerosis (n=2) with urodynamically proven neuropathic bladder dysfunction, and in controls (n=5). Inflammation and fibrosis were analysed with histological criteria and SPK1 expression was determined by individual immunohistochemical staining.

**Results:**

Significant increased SPK1 urothelial immunoreactivity was shown in patients compared to control group (p=0.03). By contrast, SPK1 immunoreactivity in patients was significantly decreased in the sub-urothelium, muscles and nerves, p=0.02; 0.01 and 0.003, respectively. Patients with neurogenic detrusor overactivity (NDO) had higher SPK1 urothelium expression than those without any DO (p=0.04).

**Conclusions:**

SPK1 is expressed in the human bladder wall, specifically the urothelium, in bladder specimens from patients with NDO. The role of SPK1 in the pathophysiology of NDO needs further elucidation.

## INTRODUCTION

Lower urinary tract dysfunction (LUTD) is common following neurological disorders such as spinal cord injury and multiple sclerosis. The commonest manifestation is an overactive bladder syndrome, characterized by urinary urgency, frequency and urge incontinence due to neurogenic detrusor overactivity (NDO). One pathophysiological hypothesis is an increase in afferent input from the bladder where nonadrenergic noncholinergic mechanisms become predominant (1). A shift from A delta-fibers to abnormal C-fibers activity is also observed. There is good evidence that the main transmitter contraction is ATP and that nitric oxid (NO) is responsible for the main part of inhibitory NANC responses (2). However, the mechanisms of altered excitability are not totally understood and the role of many others substances such as neuropeptides need to be fully established.

Sphingosine 1-phosphate (S1P) is a bioactive sphingolipid that is known to mediate diverse cellular mechanisms such as apoptosis and proliferation. These effects have been attributed to specific G protein-coupled receptors, namely S1P_1-5_ (3). In addition to its role in tumour proliferation and immunity, S1P plays an essential role in smooth muscle (3). Sphingosine kinase (SPK) 1 is one of the two major enzyme isoforms which converts sphingosine to S1P via reversible phosphorylation. In rabbits it has been reported that S1P was involved in the regulation of detrusor contractions (4, 5). In an overactive bladder rat model, greater S1P expression associated with Rho-kinase expression was noted, suggesting a role for the S1P/SPK signalling pathway in this condition (6).

To date, bladder SPK1 expression has never been studied neither in people without any bladder disease nor in human with neuropathic bladder. Our aim was to study SPK1 bladder wall expression in the human neuropathic bladder dysfunction and to determine whether it was associated with a type of bladder dysfunction.

## MATERIALS AND METHODS

Between September 2011 and June 2012 a total of 10 patients (6 females, 4 males) underwent total cystectomy and urinary diversion in our institution. The 10 patients suffered from neurogenic LUTD due to spinal cord injury (n=8) and multiple sclerosis (n=2). The indication of surgery was failure of conservative treatment resulting in refractory urinary incontinence, recurrent urinary tract infections and renal impairment and the inability to perform clean intermittent self-catheterisation (Table-1). Urinary diversion was through an ileal conduit in all cases. No postoperative complication was observed. All patients signed a consent form allowing us to do research in the bladder specimen and the local ethic committee approved the collection. Control bladder specimens (n=5) were obtained from cadaveric donors without any neurologic diseases or lower urinary tract symptoms (Table-2). All patients provided informed consent and the study was approved by the ethic committee and by the French transplantation agency (Agence de Biomédecine).

**Table 1:** Clinical and urodynamic data of the patients group.

Patient				Clinical data					USP score		Urodynamic data	
	Sex	Age	Affection	Duration	Drainage	Treatment	Complications	Incontinence	OAB	Low stream	BC	DO
	(F/H)	(years)	(MS/SCI)	(years)	(IC/SC)	(AOT/IBT)	(OAS/RUI/RI)	(score/9)	(score/15)	(score/9)	(L/N)	(Y/N)
1	F	36	MS	39	IC	AOT	UI/RUTI	3	6	9	N	Y
2	H	55	SCI	16	SC	AOT/IBT	UI	5	15	9	L	N
3	F	68	SCI	15	SC	AOT/IBT	UI/RUI	4	0	2	N	Y
4	H	73	SCI	7	SC	AOT/IBT	RUTI/RI	3	5	7	L	N
5	H	42	SCI	14	SC	IBT	RUTI/RI	1	4	9	N	N
6	F	50	SCI	10	SC	AOT	UI/RUTI	9	20	3	N	N
7	F	42	SCI	13	SC	IBT	UI	1	2	9	N	Y
8	H	33	SCI	10	SC	AOT	UI/RI	9	13	0	N	Y
9	F	59	MS	23	SC	AOT/IBT	UI/RI	2	6	9	L	Y
10	F	47	SCI	20	IC	IBT	RI	0	5	9	L	N

**MS** = Multiple Sclerosis; **SCI** = Spinal Cord Injury; **IC** = Indwelling catheter; **SC** = Supra pubic catheter; **AOT** = Antimuscarinic Oral Therapy; **IBT** = Intravesical Botulinium Toxin Injection; **UI** = urinary incontinence; **RUTI** = Recurrent Urinary tract Infections; **RI** = Renal Impairment; usp score [7] = Urinary Symptom Profile score; **OAB** = Overactive bladder; **BC** = Bladder Compliance; **L/N** = Low/Normal; **DO** = Detrusor Overactivity

**Table 2:** Clinical data of cadaveric donors.

Control	Sex	Clinical data		
	(F/H)	Age	Neurologic disease	Cause of death
		(years)	(Yes/No)	
**1**	F	48	No	Stroke
**2**	H	62	No	Acute Myocardial Infarction
**3**	F	55	No	Cardiac Insufficiency
**4**	H	79	No	Stroke
**5**	F	66	No	Acute Myocardial Infarction

### 

#### Preoperative evaluation

Before surgery, patients had a clinical and urodynamic evaluation. Lower urinary tract symptoms were assessed using the Urinary Symptoms Profile^®^ questionnaire (7). Urodynamic evaluation was performed according to the ICS recommendations (8, 9).

#### Histological examination

From each bladder, 4 full-thickness bladder samples were harvested respectively from the dome, the two lateral faces and the trigone using a punch-biopsy device (Visipunch^®^, 8mm, Huot Instruments^®^, Menomonee Falls, WI, USA) and were fixed in 4% paraformaldehyde. Histological sections of 4μm were hematoxylin-eosin stained. Pathologist examination consisted in scoring lymphocytic and plasma cell inflammatory infiltration and fibrosis and according to severity was graded as moderate (+), mild (++) and severe (+++) respectively (10).

#### Immunochemistry

All sections were deparaffinised, hydrated, boiled with 10mmol/L of citrate buffer (pH 6) for 30 min, pretreated with 0.3% H2O2 for 5 min. Then, in moist chamber at room temperature the slices were pre-incubated in PBST (Phosphate Buffered Saline 0.1%Triton) for 3x2 min and incubated with the primary antibody (polyclonal anti-SpK1 Ab diluted 1:50, Abcam™, London, United Kingdom) for 2h. All sections were incubated 30 min in secondary antibodies coupled with Horse Radish Peroxydase (Dako EnVision™ FLEX HRP, Carpenteria, CA USA). Immuno-labelling was revealed by 3’ di-amino benzidine. At the end, slices were incubated in 5min hematoxylin to show tissue morphology. Between each step sections were washed in Phosphate Buffered Saline Triton X100. Specimens were analysed with Axiophot® microscope with magnification 100 (Zeiss, Germany) by two independent observers in a “double person blind test” and they were scored using an arbitrary intensity scale: negative (0), faint/equivocal (+), moderate (++) or strong (+++) as previously used by Apostolidis (10). Percentage of each field was taken into consideration and mean value was calculated for each slide and afterwards for each case.

### Statistical analysis

The two-sided Fisher's exact t-test was used to compare the distribution of qualitative variables. Student's t-test was used to compare means between groups. For immunochemistry studies, the relationship between urodynamic data and staining intensities was analysed by the two-sided Fisher's exact t-test. Statistical significance was set at a p value<0.05.

## RESULTS

Five patients (50%) had NDO and 4 had low bladder compliance. Clinical and urodynamic data are summarized in Table-1.

## HISTOLOGICAL EXAMINATION

No specimen displayed malignancy. Inflammatory lymphocytic/plasma cell infiltration was observed in all patients specimen with predominance in the deep layers of the bladder wall. Mild or severe muscular infiltration was observed in three cases. A significant degree of fibrosis was identified in the patients compared to controls, p<0.01, predominantly in the detrusor layer. There were no significant differences in inflammation or fibrosis in the patient group regarding age, aetiology and bladder localization.

### 

#### Immunochemistry

**Figure 1:**
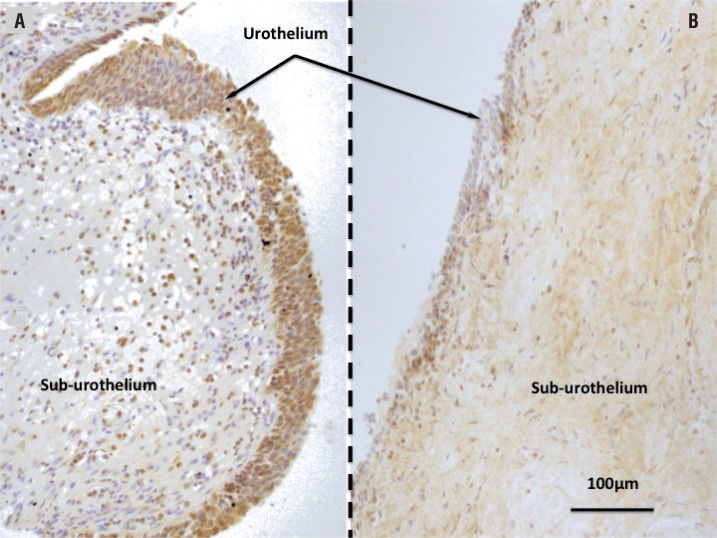
Microscopic features of sphingosine Kinase 1 expression. Transverse sections after immunochemistry reactions (Dako EnVision™ FLEX HRP, Carpenteria, CA USA). Magnification X40; scale bar=100µm. **A**) Patient with neuropathic bladder dysfunction. Significant increase in SPK1 expression is observed in the urothelium layer (arrow). **B**) Control bladder. Homogeneous expression of the Sphingosine Kinase 1 (SPK1).

**Figure 2:**
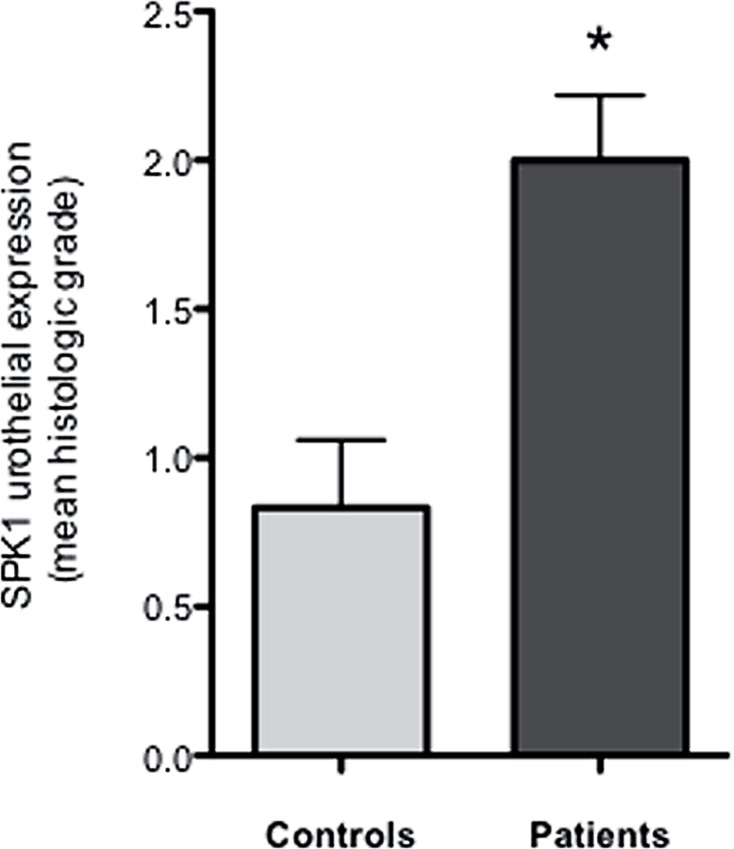
SPK1 urothelial expression. Sphingosine Kinase 1 (SPK1) expression is significantly increased in the urothelium layer of patients as compared to controls, p=0.03. * p≤0.05.

**Figure 3:**
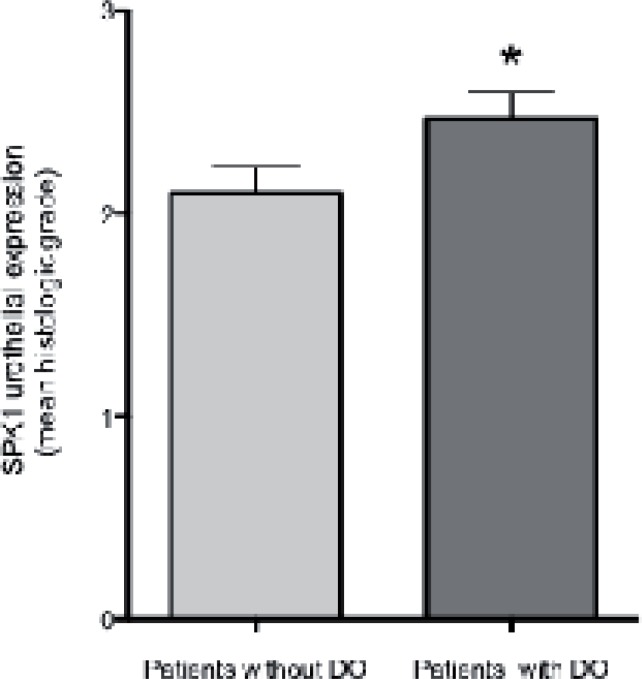
SPK1 expression and detrusor overactivity. Sphingosine Kinase 1 (SPK1) expression in bladder wall specimen of patients suffering from neuropathic bladder dysfunction. There was a significant increase in SPK1 immunoreactivity in the urothelium layer of patients suffering from detrusor overactivity (DO), p=0.04. * p≤0.05

Significantly increased SPK1 urothelial immunoreactivity was noted in patients (Figures 1 and 2) as compared to controls (p=0.03). Whereas SPK1 immunoreactivity was significantly decreased in the sub-urothelium, muscles and nerves as compared to controls, p=0.02; 0.01 and 0.003, respectively. SPK1 expression was homogeneous throughout the whole bladder in the control group (Figure-1). A significant increased in SPK1 immunoreactivity was identified in the urothelium layer of patients with DO, p=0.04 (Figure-3).

## DISCUSSION

The purpose of this study was to characterize the expression pattern of SPK1 in neurogenic LUTD. To our knowledge, this is the first time SPK1 expression has been demonstrated in human bladder tissue from patients with neuropathic bladder dysfunction. Increased expression was demonstrated in the urothelium, higher in patients with NDO as compared to patients without NDO. We confirmed also the increase in bladder wall fibrosis of patients affected by NDO which is consistent with results previously reported by Comperat et al. (11).

Recently, animal studies have attempted to understand the role of the SPK1/S1P pathway in the lower urinary tract. Watterson (4) found that S1P could contract rabbit detrusor and suggested a role of dysregulation of SPK1/S1P signalling in overactive bladder. S1P induced contraction was dependent on stretch and intracellular calcium. It was of interest that S1P was supposed to regulate calcium channels in an S1P receptor-independent manner (4).

Sandhu et al. (12) demonstrated the presence of S1P receptors in the bladder wall of female rat and found regional heterogeneity in its expression. They suggested that the significance of expression variation in the lower urinary tract could have functional and clinical involvements. They proposed this pathway as a possible therapeutic target. They demonstrated that S1P mediated smooth muscle contraction but didn't hypothesize a mechanism of inducing contraction.

In a model of overactive bladder in rat, Aydin et al. (6) reported that S1P signalling pathway was significantly up-regulated in association with overexpression of Rho kinases and they hypothesised that S1P-induced bladder overactivity. These findings may suggest that S1P modulates detrusor contraction through a Rho-kinase signalling pathway in overactive bladder.

The link between S1P and NDO is still unclear. SPK activity is known to be stimulated by agonists of various G protein-coupled receptors as well as by depolarization-induced calcium entry (13). SPK activation may in turn activate calcium sensitizing mechanisms, such as Rho-kinase signalling pathway (6), which lead to increased myosin light chain phosphorylation and detrusor contraction. Interestingly, FTY720-phosphate, an agonist for all S1P receptors except S1P_2_ induced distinct contraction properties (4). The link between S1P and detrusor contractions may involve others underlying mechanisms. S1P could act as a second messenger or interact with neurotransmitters or neuropeptides. In vascular muscles, S1P modulates muscle contraction either directly on S1P specific receptors or indirectly after being generated inside the cell (14).

Although no human data concerning SPK1 in the neuropathic bladder are available, a recent study (15) reported for the first time the presence of SPK1 and S1P receptors in the human bladder. mRNA profiling was performed on the cell lines of native human urothelium and demonstrated the expression of the two SPK isoforms SPK1, SPK2 and related receptors S1P_1-5_. No data was available concerning regional distribution and patients with neurological disorders.

Recently the urothelium has emerged more as an integrator of sensory inputs and outputs in the bladder wall than a passive barrier (16, 17). In NDO, urothelial cells exhibit “neuron like” properties that allow them to respond to various stimuli. They are believed to release many substances including acetylcholine, ATP, nitric oxide, neural growth factor and prostaglandins that can affect smooth muscle, interstitial or immune cells but that can also modulate the activity of afferent nerves (18). Recently, non-adrenergic, non-cholinergic, non-prurinergic contractions of detrusor were found in the porcine urothelium with lamina propria (19). The neurotransmitter responsible for this is still unknown. As we found a significant increase in SPK1 immunoreactivity in the urothelium as compared to control group, a potential role of S1P in this pathway cannot be excluded.

In the same way, the significant increase in SPK1 immunoreactivity in the urothelium of patients with DO as compared to patients without DO suggests that S1P/SPK signalling may play a role in human detrusor overactivity. Further studies are needed with a larger sample to confirm these findings.

The understanding of the underlying mechanism of lower urinary tract dysfunction is crucial for effective management of patients with neuropathic bladder dysfunctions. The possible involvement of SPK1/S1P pathway in human bladder dysfunction and the pathogenesis of detrusor overactivity open up a potentially novel approach to managing neurogenic LUTD. Of late, drugs modulating the SPK1/S1P pathway have been studied as disease modifying treatment in multiple sclerosis, namely the monoclonal antibody (Sphingomab® (20) and an oral S1P receptor modulator, fingolimod (21), and it would be of clinical relevance to assess the impact these drugs may have on LUTD.

The main limitation of our study was the relatively small number of tissue samples that were studied. Whilst the nature of LUTD was established in the patient group, control tissue was obtained from cadaveric donors for whom no investigation assessing bladder dysfunction was done. Lastly, S1P expression was demonstrated only by immunohistochemistry and was not confirmed using mRNA profiling. Nevertheless, our study suggests that SPK1 is expressed in the human bladder wall, specifically the urothelium, in bladder specimens from patients with NDO and additional studies are needed to confirm the role of SPK1/S1P pathway in the model of overactive bladder and a larger study is required to confirm these results and to further understand the role of this pathway in the pathogenesis of detrusor overactivity.

We investigated for the first time in a prospective study SPK1 expression in the human bladder wall and in patients suffering from neuropathic bladder dysfunction. A significant increase in its expression in the urothelium was found in all patients and it was higher in patients with detrusor overactivity. These findings suggest that SPK/ S1P pathway may be involved in the neurogenic pathological detrusor contraction mechanisms. Functional role of SPK1 is still to be confirmed. Further experiments with a larger number of patients are required to confirm highlight-underlying mechanisms. Moreover the expression in patients with idiopathic detrusor overactivity should be addressed and its level compared to patients with neurogenic detrusor overactivity.
